# Brefeldin A Reduces Anchorage-Independent Survival, Cancer Stem Cell Potential and Migration of MDA-MB-231 Human Breast Cancer Cells

**DOI:** 10.3390/molecules191117464

**Published:** 2014-10-29

**Authors:** Chao-Neng Tseng, Yi-Ren Hong, Hsueh-Wei Chang, Tsai-Jung Yu, Ting-Wei Hung, Ming-Feng Hou, Shyng-Shiou F. Yuan, Chung-Lung Cho, Chien-Tsung Liu, Chien-Chih Chiu, Chih-Jen Huang

**Affiliations:** 1 Department of Biomedical Science and Environmental Biology, Translational Research Center, Kaohsiung Medical University Hospital, Kaohsiung Medical University, Kaohsiung 80708, Taiwan; E-Mails: cntseng.tw@gmail.com (C.-N.T.); changhw@kmu.edu.tw (H.-W.C.); 2 Graduate Institute of Natural Products, Kaohsiung Medical University, Kaohsiung 80708, Taiwan; E-Mails: raynebox@yahoo.com.tw (T.-J.Y.); kva916@yahoo.com.tw (T.-W.H.); 3 Department of Biological Sciences, National Sun Yat-sen University, Kaohsiung 80424, Taiwan; E-Mail: clcho@faculty.nsysu.edu.tw; 4 Department of Biochemistry, College of Medicine, Kaohsiung Medical University, Kaohsiung 80708, Taiwan; E-Mail: m835016@cc.kmu.edu.tw; 5 Institute of Medical Science and Technology, National Sun Yat-sen University, Kaohsiung 80424, Taiwan; 6 Cancer Center and Department of Surgery, Kaohsiung Medical University Hospital, Kaohsiung 80708, Taiwan; E-Mail: mifeho@kmu.edu.tw; 7 Translational Research Center, Department of Obstetrics and Gynecology, Kaohsiung Medical University Hospital, Kaohsiung 80708, Taiwan; E-Mail: 1020005@kmuh.org.tw; 8 Department of Biotechnology, Kaohsiung Medical University, Kaohsiung 80708, Taiwan; E-Mails: u100022015@cc.kmu.edu.tw (C.-T.L.); cchiu@kmu.edu.tw (C.-C.C.); 9 Faculty of Medicine, College of Medicine, Kaohsiung Medical University, Kaohsiung 80708, Taiwan; 10 Department of Radiation Oncology, Kaohsiung Medical University Hospital, Kaohsiung Medical University, Kaohsiung 80708, Taiwan

**Keywords:** brefeldin A, cancer stem cell, ER stress, breast cancer

## Abstract

Cancer stem cells (CSCs) are a subset of cancer cells in tumors or established cancer cell lines that can initiate and sustain the growth of tumors *in vivo*. Cancer stem cells can be enriched in serum-free, suspended cultures that allow the formation of tumorspheres over several days to weeks. Brefeldin A (BFA) is a mycotoxin that induces endoplasmic reticulum (ER) stress in eukaryotic cells. We found that BFA, at sub-microgram per milliliter concentrations, preferentially induced cell death in MDA-MB-231 suspension cultures (EC_50_: 0.016 µg/mL) compared to adhesion cultures. BFA also effectively inhibited clonogenic activity and the migration and matrix metalloproteinases-9 (MMP-9) activity of MDA-MB-231 cells. Western blotting analysis indicated that the effects of BFA may be mediated by the down-regulation of breast CSC marker CD44 and anti-apoptotic proteins Bcl-2 and Mcl-1, as well as the reversal of epithelial-mesenchymal transition. Furthermore, BFA also displayed selective cytotoxicity toward suspended MDA-MB-468 cells, and suppressed tumorsphere formation in T47D and MDA-MB-453 cells, suggesting that BFA may be effective against breast cancer cells of various phenotypes.

## 1. Introduction

Mounting evidence indicates that most types of cancer, including breast cancer, originate from a small subset of cancer stem cells (CSCs) [1,2,3,4]. These CSCs are able to self-renew and give rise to other cancer cells that form a tumor mass [5,6]. These cells are believed to be responsible for tumor initiation, progression, metastasis, resistance to chemotherapy and radiation, as well as tumor relapse after therapy [7,8,9]. Therefore, targeting CSCs is critical for effective treatment of cancer. Breast cancer stem cells can be established from patients’ surgical specimens or established breast cancer cell lines based on their abilities to resist cell-detachment-induced apoptosis (anoikis) and to propagate as floating tumorspheres in suspension cultures [10].

Breast cancer tumorspheres show an increase in CSC population and are characterized by the expression of CD44^+^CD24^low/^^−^ phenotype compared to monolayer adhesion cultures; moreover, they display high tumorigenic potential in mice explant models [11]. Using suspension cultures of breast cancer cells as a drug screening platform, a number of anti-CSC drugs reagents, including natural products, that can reduce the survival of CSCs in suspension cultures and inhibit tumorsphere growth have been identified [12,13,14].

Brefeldin A (BFA) is a lactone antibiotic first isolated from the fungus *Eupenicillium brefeldianum* [15]. BFA inhibits the transport of secreted and membrane proteins from endoplasmic reticulum (ER) to Golgi apparatus, leading to disruption of Golgi function, accumulation of unfolded and not fully incompletely processed proteins in ER and, finally, the induction of ER stress [16]. Prolonged ER stress could result in cell death through classical ER stress-signaling pathways originating from ER-residing proteins, PERK/eIF2*α*, IRE1α/XBP1 and ATF6, and their downstream mediators such as C/EBP homologous protein (CHOP) [17,18].

Previously, we reported the cancer-inhibitory ability of BFA in colon cancer [13]. Here we investigated the effects of BFA on the CSC properties of MDA-MB-231 human breast cancer cells and the potential underlying mechanism.

## 2. Results and Discussion

### 2.1. BFA Induces Anchorage-Independent Cell Death in MDA-MB-231 Breast Cancer Cells

**Figure 1:**
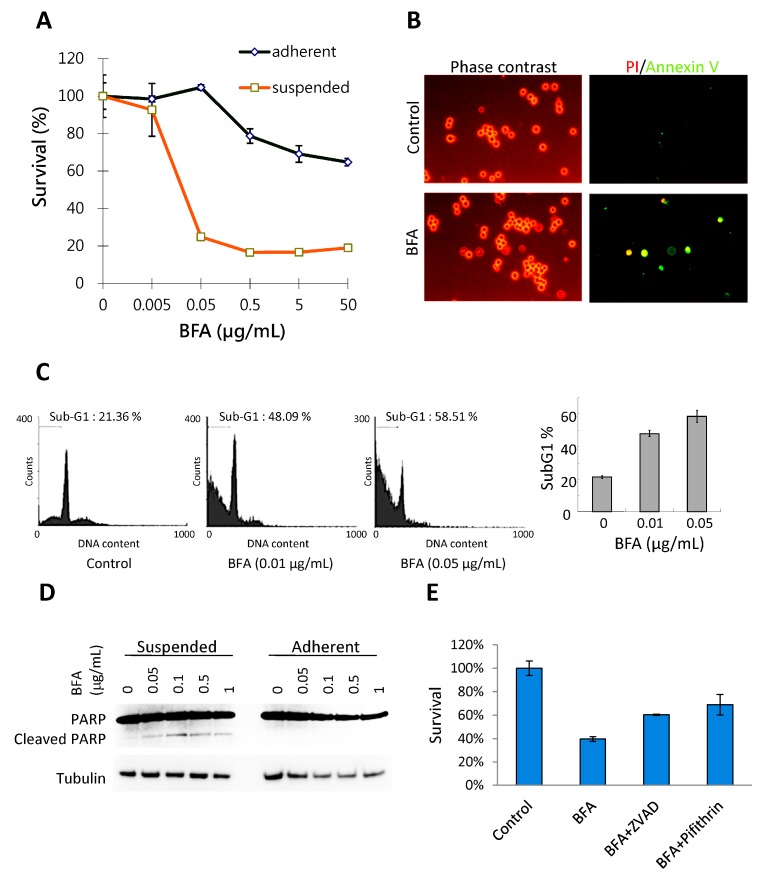
BFA induced anchorage-independent cell death in MDA-MB-231 breast cancer cells. (**A**) Survival curves of suspended or adherent MDA-MB-231 cells treated with 0–50 μg/mL BFA for 48 h as determined by WST-1 assay (mean ± SD, n = 3) (**B**) PI^−^/Annexin V^+^ apoptotic cells can be detected in suspension cultures treated with 0.1 μg/mL BFA for 4 h under a fluorescent microscope. (**C**) Flow cytometric analysis indicated that BFA treatments at 0.01 and 0.05 μg/mL for 24 h increased the sub-G1 fraction of suspended MDA-MB-231 cells from 22%± 1.34% to 47%± 2.71% and 58.52%± 3.32%, respectively. (mean ± SD, n = 3). (**D**) Cleaved PARP fragments could be detected by Western blotting in suspended MDA-MB-231 cells treated with 0.05–1 μg/mL BFA for 24 h but not in adherent cells. (**E**) Pretreatment with 100 μM Z-VAD-FMK or 20 μM Pifithrin-α increased the survival of cells in the presence of 0.05 μg/mL BFA from 40.16%± 0.83% to 61.05%± 0.57% and 74.52%± 5.34%, respectively.

Anoikis resistance is considered an essential step in tumor metastasis [19]. The effects of BFA on the growth of MDA-MB-231 human breast cancer cells were tested both in adhesion and suspension cultures. MDA-MB-231 cells were treated with 0–50 μg/mL BFA for 48 h and the survival curves were determined by WST-1 assay. BFA, at concentrations higher than 0.5 μg/mL, slightly inhibited the growth of adherent cells to 80%–60% of control, whereas suspension cells were extremely sensitive to BFA with survival dropping to about 20% in the presence of 0.05–50 μg/mL BFA (Figure 1A). EC_50_ was determined to be 0.016 µg/mL using GraphPad Prism. Live cell staining with propidium iodide (PI) and Annexin V indicated that PI^−^/Annexin V^+^ early apoptotic cells can be detected in suspension cultures exposed to 0.1 μg/mL BFA for 4 h (Figure 1B). Flow cytometric analysis also indicated that treating suspended MDA-MB-231 cells with 0.01 and 0.05 μg/mL BFA for 24 h drastically increased the fraction of sub-G1 cell debris (Figure 1C). PARP (poly ADP-ribose polymerase-1) cleavage, a signature event of cell death, could be detected by western blotting in suspended MDA-MB-231 cells treated with 0.05–1 μg/mL BFA for 24 h (Figure 1 D). The faint PARP cleavage product in control cells was likely due to that a small portion of cells failed to adapt to the suspension culture condition in time and succumbed to apoptosis. Pretreatment with 100 μM Z-VAD-FMK, a pan-caspase inhibitor, or 20 μM pifithrin-α, a p53 inhibitor, increased the survival of cells in the presence of 0.05 μg/mL BFA, suggesting that BFA induced apoptosis in suspended MDA-MB-231 cells and that p53 may be involved.

### 2.2. BFA Inhibits the Formation of MDA-MB-231 Colonies in 3D and 2D Cultures

**Figure 2:**
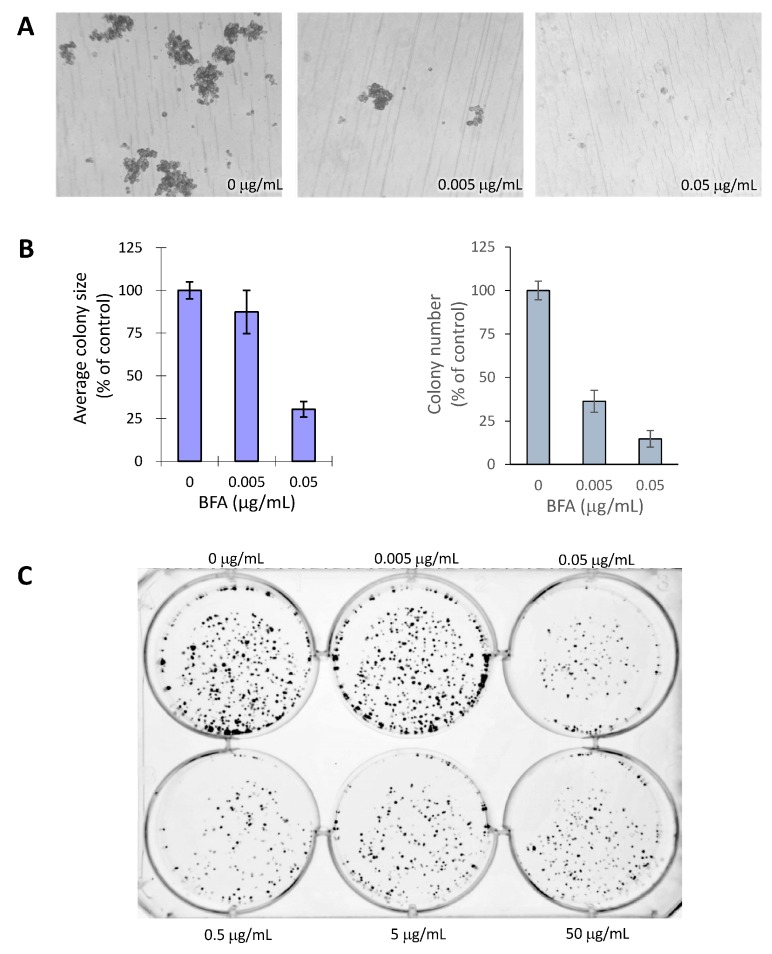
BFA inhibited the formation of 3D and 2D colonies in MDA-MB-231. (**A**) Representative micrographs showing the formation of tumorspheres by MDA-MB-231 cells treated with 0, 0.005 and 0.05 μg/mL BFA for 7d using soft agar assay. (**B**) Quantitative analyses of results from (A) using ImageJ software show the reduction of the size and number of tumorspheres by BFA. (**C**) Colony formation by adherent MDA-MB-231 cells pretreated with 0–50 μg/mL BFA for 24 h. Colonies were generated from 1000 viable cells per well, allowed to grow for 12 d, and then fixed and stained by crystal violet.

A hallmark of the cancer stem cell is its ability to generate colonies from isolated single cells in suspension [20]. However because MDA-MB-231 cells of the same colony do not aggregate well and easily drift away in suspension cultures making it difficult to outline each colony, we chose 3D colony formation in soft agar assay which is also linked to MDA-MB-231 cancer stem cell activity [1,2,3]. We first tested if treatments with 0, 0.005 and 0.05 μg/mL BFA for 7 d could inhibit the formation of tumorspheres from individual cells suspended in soft agar. Results indicated that the size and number of MDA-MB-231 tumorspheres were reduced by BFA to different degrees (Figure 2A,B). At 0.005 μg/mL, BFA significantly reduced the number of tumorspheres but not their sizes, suggesting BFA probably reduced the number of cancer stem cells but was less effective in inhibiting the growth of their daughter cells. We also tested the effect of BFA on the clonogenic potential of MDA-MB-231 by pretreating adherent MDA-MB-231 cells with 0–50 μg/mL BFA for 24 h and allowing colonies to be generated from 1000 viable cells per well in a 6-well plate for 12 d. The results showed that pre-treating adherent MBA-MB-231 cells with ≥0.05 μg/mL BFA clearly reduced the fraction of cells with clonogenic potential (Figure 2C).

### 2.3. BFA Inhibits the Migration and MMP 9 (Matrix Metallopeptidase 9) Activity of MDA-MB-231

**Figure 3:**
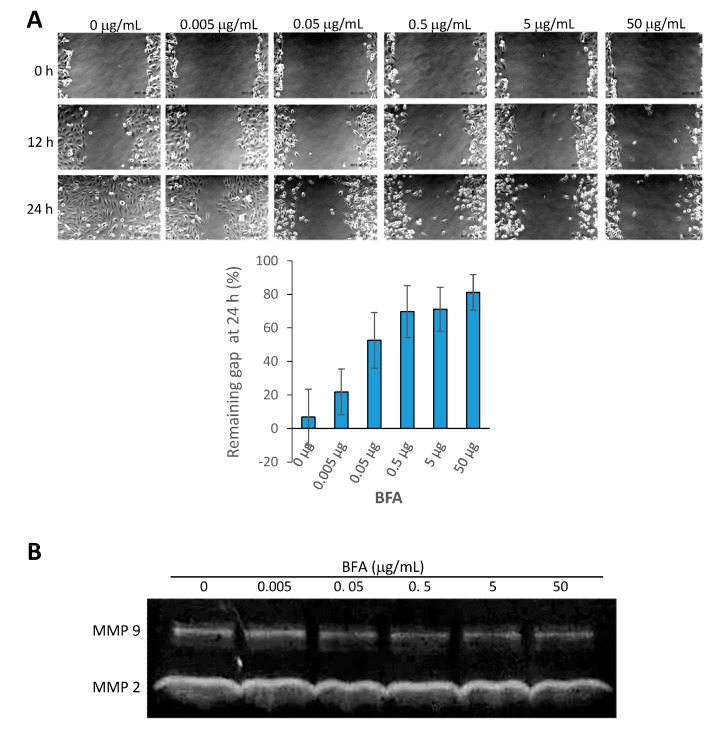
BFA inhibited the migration and MMP 9 activity of MDA-MB-231. (**A**) Wound healing assay was used to assess the inhibitory effect of BFA on MDA-MB-231 migration. Quantitative analysis showed BFA at concentrations ≥0.05 μg/mL significantly increased the remaining gap at 24 h. (**B**) MMP activities in the concentrated conditional media from MDA-MB-231 cells treated with 0–50 μg/mL BFA for 24 h were assessed by gelatin zymography. The gelatin-free zone created by MMP 9 became smaller in the presence of BFA.

MMP activity is increased in breast cancer and stimulates tumorigenesis [21]. In addition to being a downstream target of EMT regulators, MMP-9 is able to promote EMT in corporation with Snail [22]. The cancer stem cell marker CD44 has been shown to serve as a cell surface receptor/substrate for MMP-9 to enhance cancer cell migration and tumor invasion [23,24]. Previous studies reported by us and others show that cancer stem cell inhibitors usually are also capable of suppressing the metastasis potential of cancer cells, such as migration ability and MMP activities [13,25,26]. Here, we found that 24 h treatment of BFA at concentrations ≥0.05 μg/mL significantly retarded the migration of MBA-MB-231 cells in the wound healing assay (Figure 3A). In addition, gelatin zymography indicated that BFA reduced the activity of MMP 9, but not MMP2, secreted by MDA-MB-231 (Figure 3B).

### 2.4. Modulation of Protein Expression by BFA

**Figure 4:**
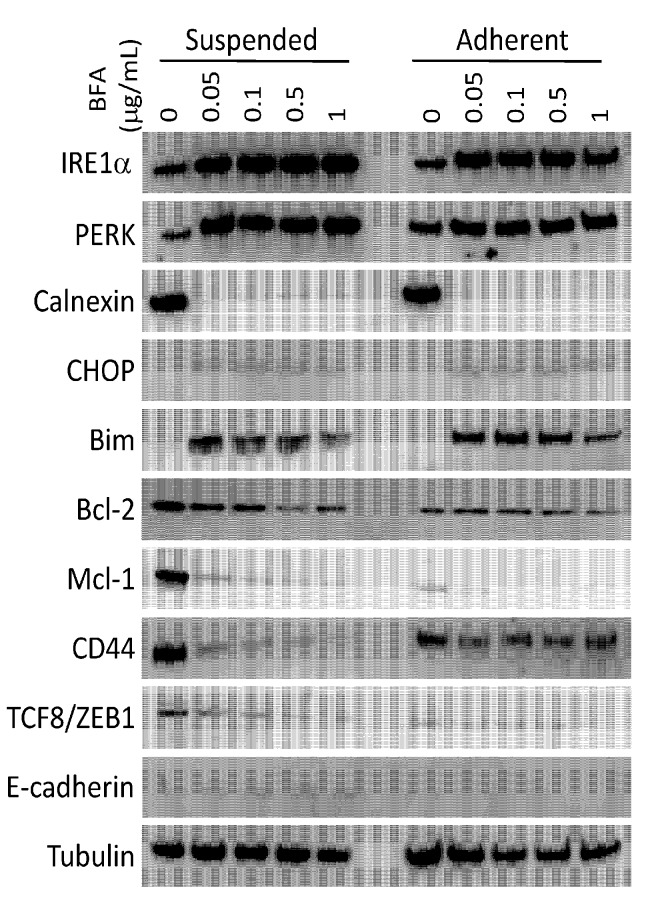
Modulation of protein expression by BFA. The total cell lysates of suspended or adherent MDA-MB-231 cells treated with 0–1 μg/mL for 24 h were analyzed by western blotting to examine the expression of ER stress response proteins (IRE1α, PERK, Calnexin and CHOP), the BCL family apoptosis regulators (Bim, Bcl-2 and Mcl-1), proteins involved in epithelial-mesenchymal transition (TCF8/ZEB1 and E-cadherin), and CD44.

To explore the underlining mechanism responsible for the effects of BFA, we examined the expression of selected proteins involved in ER stress response, apoptosis regulation, epithelial-mesenchymal transition, and maintenance of cancer stem cell properties by western blotting (Figure 4).

ER stress response was activated by 24 h treatment with BFA at all tested concentrations in both adherent and suspended MDA-MB-231 cells, indicated by the up-regulation of IRE1α, PERK and CHOP, and the abrupt down-regulation of calnexin, although PERK was up-regulated to a stronger degree in suspended cells. Bim, a pro-apoptotic BCL-2 family protein, was readily activated by BFA at concentrations as low as 0.05 μg/mL BFA. Two pro-survival BCL-2 family proteins, Bcl-2 and Mcl-1, were down-regulated by BFA. At higher BFA concentrations (0.5~1 μg/mL), defective ER may start to affect protein synthesis directly or indirectly. Up regulation of Bim and Bcl-2 protein expression by BFA therefore became less prominent. Notably, Mcl-1, which has been reported to be critical for cancer cells to undergo anchorage-independent growth, was highly expressed in suspended MDA-MB-231 cells. A previous study found that dysregulation of Mcl-1 degradation and Bim induction during detachment contributes to decreased anoikis sensitivity of metastatic cells [27]. The decrease of Mcl-2 protein expression by BFA therefore may be a key to the preferential cytotoxicity of BFA toward suspended MDA-MB-231 cells. TCF8/ZEB1, a transcription factor that promotes EMT and tumor progression [28,29], was expressed in suspended MDA-MB-231 cells and was down-regulated by BFA. Down-regulation of TCF8/ZEB1 may independently contribute to BFA-induced anoikis, since knockdown of TCF8/ZEB1 has been reported to suppress anchorage-independent cell growth of lung cancer cells [30]. In accord with the down-regulation of TCF8/ZEB1, BFA up-regulated the expression of E-cadherin, a cell-cell adhesion protein expressed mainly in epithelium and whose suppression by TCF8/ZEB1 is one of the hallmarks of EMT and late-stage cancer progression [31,32]. The breast cancer stem cell marker CD44 was down-regulated by BFA in both suspended and adherent cultures but to a lesser degree in adherent cells. CD44 is a multi-functional protein involved in cell migration [33], growth factor signaling [34], and organization of actin cytoskeleton [35]. Therefore, these results are in line with the multiple effects of BFA in reducing anchorage-independent survival, cancer stem cell potential and migration capability of MDA-BA-231 cells. Whether BFA reduces cancer stem cell population requires detail cytometric analysis of the expression of additional cancer stem cell markers.

### 2.5. Inhibitory Effects of BFA on MDA-MB-468, T47D, and MDA-MB-453 Human Breast Cancer Cells

**Figure 5:**
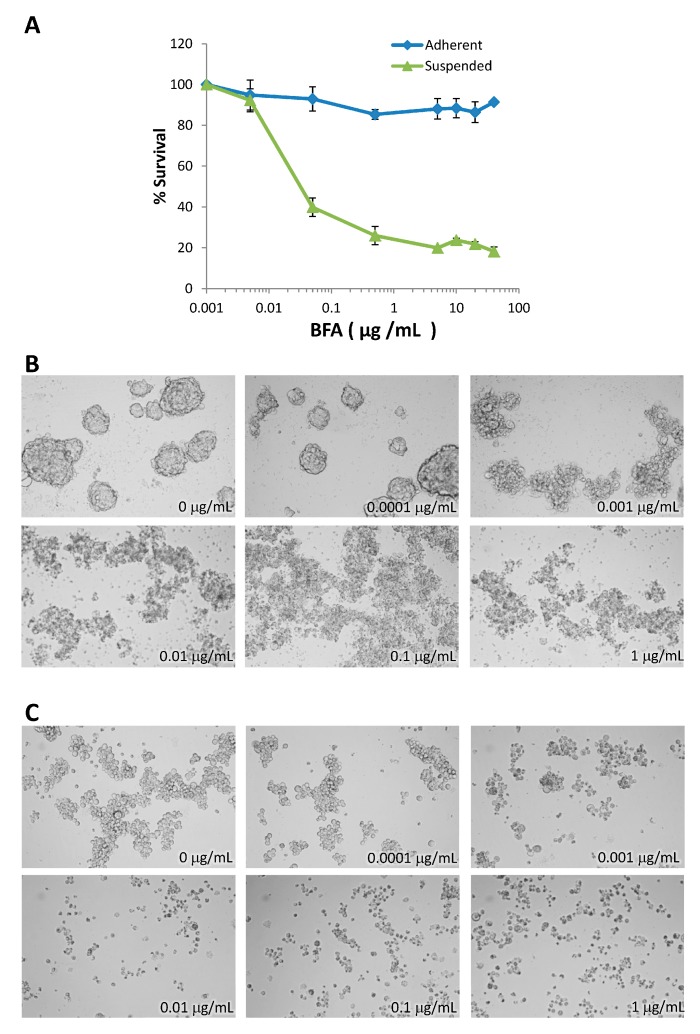
Inhibitory effects of BFA on MDA-MB-468, T47D, and MDA-MB-453 human breast cancer cell lines. (**A**) BFA displayed selective cytotoxicity toward suspended MDA-MB-468 cells. Tumorsphere cultures (7 d) of T47D (**B**) and MDA-MB-453 (**C**) treated with 0–1 μg/mL BFA photographed under 10 X phase contrast microscopy.

To test if the effects of BFA are limited to MDA-MB-231, we also tested three other human breast cancer cell lines: MDA-MB-468 (ER^−^/PgR^−^/ERBB2^−^, same as MDA-MB-231), T47D (ER^+^/PgR^+^/ERBB2^−^), and MDA-MB-453 (ER^−^/PgR^−^/ERBB2^+^). This assessment indicated that BFA also displayed selective cytotoxicity toward suspended MDA-MB-468 cells (Figure 5A), and it also suppressed tumorsphere formation in T47D (Figure 5B) and MDA-MB-453 (Figure 5C), suggesting that BFA may be effective against breast cancer cells of various phenotypes.

### 2.6. Discussion

The cytotoxic effect of BFA in suspension cells already reaches maximum at 0.05 μg/mL (Figure 1A). One would expect the signaling pathway mediating the specific effect of BFA is also activated to the full extent by 0.05 μg/mL BFA. Coincidentally, there is no clear dose-dependent change of PARP cleavage (Figure 1D), clonogenic activity (Figure 2C), and MMP9 activity (Figure 3B) once BFA concentration was higher than 0.05 μg/mL. These results may suggest that these diverse effects of BFA are mediated by a common signaling pathway.

BFA is typically used at 2–5 μg/mL in ER stress studies. Here we show that ER stress can be induced by BFA at much lower concentrations. Since the sensitivity of suspended MDA-MB-231 to BFA is a novel finding, this study is our first attempt to compare BFA-induced ER stress response in MDA-MB-231 under suspension and adherent conditions. Based on Western blotting analysis of representative signaling proteins of ER stress pathway, this pathway seems to be activated to the same degree in suspended and adherent cultures. We suspect that differential expression of apoptosis regulators (e.g., Mcl-1) in these two types of cultures may determine the sensitivity of MDA-MB-231 cells to ER stress-induced cell death.

Since the concentration of BFA required to sensitize suspended MDA-MB-231 to cell death is very low, we believe it is unlikely to have significant effects to basic cellular functions. Besides, effects such as global inhibition of protein translation, if induced by BFA at such low concentrations, should also have similar impact in adherent cells. Nevertheless, cancer stem cells rely heavily on trophic factors through autocrine [36,37] and paracrine [38,39] pathways that require the synthesis of membrane receptors and secretory factors from ER. Whether these signals are easily hampered by BFA requires further investigation.

## 3. Experimental Section

### 3.1. Adhesion and Suspension Cell Cultures

Cells were cultured in Dulbecco’s modified Eagle medium (DMEM) supplemented with 10% fetal calf serum and 0.1 mM nonessential amino acids at 37 °C in a humidified atmosphere with 5% CO_2_. For CSC culture and the formation of tumospheres, detached cells were suspended in DMEM–F12 with 1X B27 supplement, 0.4% bovine serum albumin, 5 μg/mL bovine insulin, 20 ng/mL basic fibroblast growth factor 2, and 10 ng/mL epidermal growth factor at a density of 1000 cells/mL in ultralow attachment 96 well plates. For prolonged culture of tumorspheres, fresh media were replenished every 3 days.

### 3.2. Trypan Blue Cellular Viability Assessment

Cellular viability was determined using the trypan blue dye exclusion method (final dye concentration: 0.04% *w*/*v*) and a hemocytometer.

### 3.3. WST-1 Cell Survival Assay

WST-1 reagent (Roche, Mannheim, Germany) was added to cells in a 1:10 dilution and incubated for 4 h. The absorbance at 450 nm of each well was measured and normalized to the average reading of control cells.

### 3.4. Flow Cytometic Analysis of Sub-G1 Population and Live Cell Annexin V/PI Staining

For the determination of sub-G1 fraction by flow cytometry, cells were trypsinized, washed with PBS and re-suspended in hypotonic lysis solution (100 mM Tris, 154 mM NaCl, 1 mM CaCl2 and 0.5 mM MgCl2 and 0.1% NP-40) with 50 μg/mL of propidium iodide (PI) and 40 μg/mL of RNase. The cells were incubated on ice for 20 min before flow cytometry analysis. To detect early apoptotic cells, un-fixed cells were washed by PBS and stained with Annexin V-FITC and PI using AnnexinV-FITC Apoptosis Detection Kit (Strong Biotech, Taipei, Taiwan).

### 3.5. 3D Soft Agar Tumorsphere Formation Assay

Cells (5 × 10^4^) were carefully mixed with 2% molten low melting agarose in DMEM–F12 growth medium to reach a final concentration of 0.4% agarose. The cell-agarose mixture was then placed on top of a solidified layer of 0.5% agarose with 1× growth medium in the six-well plate. Cells in the upper layer were fed with growth medium every 6 to 7 days.

### 3.6. 2D Clonogenic Assay

After pretreatment with 0–50 μg/mL BFA for 24 h, the cells were reseeded at a density of 1 × 10^3^ cells per well in the 6-well plate and cultured for additional 12 days, with medium changed every 3 days. The colonies were then fixed for 15 min with methanol-acetic acid (3:1) and stained with crystal violet (1%) for 30 min at room temperature.

### 3.7. Wound-Healing Motility Assay

Scratch wounds were created using a p10 micropipette tip in six-well plates with overnight confluent cultures. After cell debris were washed three times with phosphate-buffered saline (PBS), cells were replenished with complete medium containing 0–50 μg/mL BFA. Images of wound healing were captured by phase-contrast microscopy at indicated times after wounding.

### 3.8. Gelatin Zymography

Supernatants were collected from cell cultures treated with 0–50 μg/mL BFA for 24 h, filtered through a 0.22 μm filter, and concentrated 50 X by using Centricon spin columns with 10 kD cutoff. Concentrated supernatants were resolved on non-reducing SDS-PAGE using 10% polyacrylamide gels containing 0.1% SDS and 1 mg/mL gelatin. After electrophoresis, the gels were washed three times with 50 mM Tris-HCl (pH 7.5) containing 0.15 M NaCl, 5 mM CaC1_2_, 5 μM ZnCl, 0.02% NaN_3_, 0.25% Triton X-100 at room temperature for 30 min each time, and then the gels were incubated in the same buffer without Triton X-100 at 37 °C for 20 h. Coomassie Brilliant Blue R-250 staining was used to reveal gelatin-clear zones created by MMPs.

### 3.9. Western Blotting

Western blotting was performed using Bolt^®^ 4%–12% Bis-Tris Plus Gels with Bolt^®^ Mini Gel Tanks, Bolt™ Mini Blot Modules, and iBind™ Western Systems from Life Technologies (Carlsbad, CA, USA) according to the manufacturer’s instructions. Antibodies used in this study were: PARP Antibody #9542, IRS1α (14C10) antibody #3294, PERK (D11A8) antibody #5683, Calnexin (C5C9) antibody #2679, CHOP (L63F7) antibody #2895, Bim (C34C5) antibody #2933, Bcl-2 (50E3) antibody #2870, Mcl-1 (D35A5) antibody #5453, TCF8/ZEB1 (D80D3) antibody #3396, E-cadherin (24E10) antibody #3195 from Cell Signaling Technology (Danvers, MA, USA); Anti-CD44 [H-CAM] Antibody, clone EPR1013Y from Millipore (Billerica, MA, USA); β-Tubulin Loading Control Antibody (4D11) from ThermoFisher Scientific (Waltham, MA, USA).

### 3.10. Statistical Analysis

All experiments were repeated at least three times. Results are presented as mean ± SD unless stated otherwise. Data were analyzed with Student’s *t*-test. *p* values < 0.05 were considered statistically significant. Single and double asterisks denote *p* < 0.05 and *p* < 0.01, respectively.

## 4. Conclusions

BFA exerts multiple effects including inducing anoikis, reducing cancer stem cell activities, and inhibiting migration ability in human breast cancer MDA-MB-231 cells. Western blotting analysis indicated that these effects of BFA may be mediated by the down-regulation of breast CSC marker CD44 and anti-apoptotic proteins Bcl-2 and Mcl-1, as well as the reversal of epithelial-mesenchymal transition.
